# Nanocomplexes of Graphene Oxide and Platinum Nanoparticles against Colorectal Cancer Colo205, HT-29, HTC-116, SW480, Liver Cancer HepG2, Human Breast Cancer MCF-7, and Adenocarcinoma LNCaP and Human Cervical Hela B Cell Lines

**DOI:** 10.3390/ma12060909

**Published:** 2019-03-19

**Authors:** Marta Kutwin, Ewa Sawosz, Sławomir Jaworski, Mateusz Wierzbicki, Barbara Strojny, Marta Grodzik, Malwina Ewa Sosnowska, Maciej Trzaskowski, André Chwalibog

**Affiliations:** 1 Department of Animal Nutrition and Biotechnology, Faculty of Animal Science, Warsaw University of Life Sciences, 02-786 Warsaw, Poland; ewa_sawosz@sggw.pl (E.S.); slawomir_jaworski@sggw.pl (S.J.); mateusz_wierzbicki@sggw.pl (M.W.); barbara_strojny@sggw.pl (B.S.); marta_grodzik@sggw.pl (M.G.); malwina_sosnowska@sggw.pl (M.E.S.); 2 Centre for Advanced Materials and Technologies CEZAMAT, Warsaw University of Technology, 02-822 Warsaw, Poland; m.trzaskowski@cezamat.pl; 3 Faculty of Chemical and Process Engineering, Warsaw University of Technology, 00-645 Warsaw, Poland; 4 Department of Veterinary and Animal Sciences, Faculty of Health and Medical Sciences, University of Copenhagen, 1870 Frederiksberg, Denmark; ach@sund.ku.dk

**Keywords:** cancer, platinum nanoparticles, graphene oxide, nanocomplex

## Abstract

Inefficient drug administration into cancer cells is related to the chemoresistance of cancer cells caused by genetic mutations including genes involved in drug transport, enzyme metabolism, and/or DNA damage repair. The objective of the present study was to evaluate the properties of platinum (NP-Pt), graphene oxide (GO), and the nanocomplex of GO functionalized with platinum nanoparticles (GO-NP-Pt) against several genetically, phenotypically, and metabolically different cancer cell lines: Colo205, HT-29, HTC-116, SW480, HepG2, MCF-7, LNCaP, and Hela B. The anticancer effects toward the cancer cell lines were evaluated by 2,3-bis-(2-methoxy-4-nitro-5-sulfophenyl)-2H-tetrazolium-5-carboxyanilide salt (XTT) and bromodeoxyuridine (BrdU) assays and measurements of cell apoptosis and morphology deformations. The NP-Pt and GO could effectively be introduced to cancer cells, but more effective delivery was observed after GO-NP-Pt treatment. The delivery of the GO-NP-Pt nanocomplex significantly decreased the viability of Colo 205 and HepG2 cells, but did not increase the cytotoxicity of other investigated cancer cells. The nanocomplex GO-NP-Pt also significantly increased the apoptosis of Colo 205 and HepG2 cancer cells. The obtained results suggest that the nanocomplex GO-NP-Pt is a remarkable nanostructure that can improve the delivery of Pt nanoparticles into cancer cells and has potential anticancer applications.

## 1. Background

Abnormalities of cell structures and their functions are caused by external factors such as ultraviolet (UV) light, bacterial and virus infections, as well as obesity and an unhealthy lifestyle [1]. The formation of cancer cells is also related to genetic mutations and abnormalities in hormone and immune conditions [2]. These factors are responsible for cancer development, but also have an influence on the chemoresistance of cancer cells. The standard procedures of cancer treatments are based on surgical resection, radiotherapy, and chemotherapy. Despite increasing research efforts, the knowledge of the main mechanisms of developing chemoresistance in different types of cancer cells is not commonly used in clinical oncology. Inefficient drug administration into cancer cells is related to cancer resistance due to genetic mutation, including genes involved in drug transporting, metabolizing enzymes, and DNA damage repair [3]. 

Nanobiotechnology has introduced a new perspective for using nanosized elements against cancer diseases. Nanoparticles, because of their size (<100 nm), have unique physiochemical features including a large surface-to-mass ratio, easy surface functionalization, quantum characteristics [4,5], and, consequently, novel biological properties. The cellular transport of nanoparticles is also significantly different from that of chemical compounds or drugs because they are able to move across cells, reach nuclear membranes, and target specific structures such as proteins or gene sequences [6,7]. Nanoparticles of graphene oxide (GO), the thinnest material, with the thickness of one carbon atom, have recently been evaluated as a useful biomaterial for cancer therapy and diagnosis. The GO nanosheets produced by the modified Hummer´s method are stable in water suspensions, mainly because of the presence of hydrophilic oxygenated groups on their surface as well as on the edges [7]. According to a previous study, GO has a minor effect on the viability and morphology of different cells, including glioma U87 and U118 cells [8]. On the other hand, Chang et al. [9] reported that GO may generate oxidative stress, causing adverse effects on the viability of A549 cancer cells. The GO treatment led to the structural deformation of mitochondria, affecting the mitochondria membrane potential [10]. Moreover, other examples of nanoparticles like gold nanoparticles, of which clinical application in head and neck cancer treatment was accepted by the FDA [11], also showed in other studies harmful effects on mammal cell toxicity and induced long-term organ damage [12]. However, because of minor harmful effects on cell viability, GO has been investigated [13] as a potential delivery platform for drugs, bioactive molecules, DNA, RNA, and other oligonucleotides [14]. 

The catalytic properties of platinum atoms are useful for cancer treatment. However, the lack of selectivity between cancer and non-cancer cells is the major drawback of Pt therapy [15]. Moreover, the development of chemoresistance by cancer cells is the second largest challenge for treatment strategies. The use of Pt-based nanomaterials can be a solution for some of the side effects of platinum-based cancer therapy. Platinum nanoparticles (NP-Pt) have catalytic activity and a high reactivity, similar to Pt-based drugs, but their large surface-to-mass ratio is a unique physiochemical feature and have an impact on their size-dependent bioavailability [15]. Previous studies have demonstrated that NP-Pt has anticancer activity against several cancer cell lines, e.g., glioma U87 [16,17] and U251 [18], colorectal HT29 [19], breast MCF-7, liver HepG-2 [20], and lymphoma U937 [21]. The genotoxic effect of NP-Pt was also observed in non-cancer cell lines, human bronchial epithelial cells HBEC3-kt, where NP-Pt increased micronucleus and DNA degradation [22]. The NP-Pt mechanism of apoptosis activation is based on the direct interaction of Pt with the DNA of cancer cells [16,17,18,20] and additionally by Pt^+^ ions released under H_2_O_2_ generation in endosomes [15]. Brown et al. [23] also showed that after intravenous injection of NP-Pt into mice with the HepG2 liver tumor, NP-Pt affected the tumor tissue, but were also accumulated in spleen and liver tissue. Moreover, NP-Pt circulating in the bloodstream were detected, which means that NP-Pt are able to cross membrane barriers and cause adverse effects. Zhang et al. [24] showed that NP-Pt were efficiently deposited on the GO surface but did not show the anticancer activity against MCF-7 and SGC-7901 cell lines. We hypothesized that the use of GO as a carrier for Pt-NP can retain NP-Pt directly on the cancer cell surface, slow the release of Pt into cells, limit the possibility of uncontrolled circulation of NP-Pt, and, consequently, increase the anticancer efficiency. In this context, the objective of this study was the characterization of anticancer properties of GO-NP-Pt against morphologically, metabolically, and genotypically different cancer cell lines. 

## 2. Materials and Methods

### 2.1. Preparation and Characterization of Nanocolloids

#### 2.1.1. Platinum Nanoparticles and Graphene Oxide

The colloid of platinum nanoparticles was purchased from Nano-koloid (Warsaw, Poland). This material is produced by a patented electric non-explosive method (Polish patent 380649) from high-purity metal (99.9999%) and high-purity demineralized Milli-Q water. Graphene oxide powder (GO) (purity 99.99%) was purchased from CEZEMAT (Warsaw, Poland) and dispersed in ultrapure water to prepare a 1.0 mg/mL solution.

After 30 minutes of sonification, the hydrocolloids of GO-NP-Pt, NP-Pt, and GO were diluted to different concentrations with 1× Dulbecco’s modified Eagle’s culture medium (Sigma-Aldrich, St. Louis, MO, USA) immediately prior to exposure to cells.

#### 2.1.2. Complex of Graphene Oxide and Platinum Nanoparticles

Suspensions of GO (200 μg/mL) and NP-Pt (100 μg/mL) were prepared in ultrapure water and used without additional purification and filtration. Ultrasonic coating of GO nanosheets for 30 min took place in a 50-mL glass flask.

#### 2.1.3. Scanning Electron Microscopy

The shape and size of the NP-Pt GO and GO-NP-Pt complexes were inspected using a scanning transmission electron microscope. Electron microscope images were taken with the use of a Hitachi SU8230 ultra-high-resolution field emission scanning electron microscope (Hitachi High-Technologies Corporation, Tokyo, Japan), using the transmission mode at 30.0 kV accelerating voltage. Images were taken with the use of gold transmission electron microscopy (TEM) grids coated with Lacey carbon film, which were immersed in GO samples and dried prior to observation.

#### 2.1.4. Fourier Transform Infrared (FTIR) Spectroscopy

The FTIR spectra of dry samples of NP-Pt, GO, and GO-NP-Pt were recorded using a Nicolet 6700 FTIR spectroscope with diamond ATR pickup (Thermo Scientific, Waltham, MA, USA). All samples were prepared by dropping 500 µL of sample suspension on a microscope glass and drying the suspension.

#### 2.1.5. ζ-Potential Measurements

The ζ-potentials of NP-Pt, GO, and GO-NP-Pt were measured by the laser dynamic scattering electrophoretic method, using the Smoluchowski approximation with a Zetasizer Nano ZS90 (Malvern Instruments, Malvern, UK). Each sample was measured after stabilization at 25 °C for 120 s. All measurements were performed in triplicate.

### 2.2. Cell Cultures and Treatments

Based on previous unpublished data and cancer statistic [25] the different cancer cell lines were selected for in vitro investigation. The cell lines MCF-7, LNCaP, Hela B, HepG2, SW480, HT29, HCT116, and Colo205 were obtained from the American Type Culture Collection (Manassas, VA, USA) and maintained in Dulbecco’s modified Eagle’s (MCF-7, Hela B, HepG2), Roswell Park Memorial Institute (LNCaP, Colo205, SW480), or McCoy’s 5A culture medium (HT29, HCT116), supplemented with 10% fetal bovine serum (Sigma-Aldrich), 1% penicillin, and streptomycin (Sigma-Aldrich) at 37 °C in a humidified atmosphere of 5% CO_2_/95% air in a NuAire DH AutoFlow CO_2_ Air-Jacketed Incubator (Plymouth, MN, USA).

### 2.3. Proliferation Assay

Cell proliferation was evaluated using the bromodeoxyuridine (BrdU) incorporation assay (BrdU colorimetric) (Roche Applied Science, Indianapolis, IN, USA). The cell lines MCF-7, LNCaP, Hela B, HepG2, SW480, HT29, HCT116, and Colo205 (1 × 10^4^) were placed in 96-well plates and cultured overnight. Next, the medium was removed and hydrocolloids of GO at concentrations of 5.0, 10.0, 25.0, 50.0, and 100.0 µg/mL, NP-Pt at concentrations of 0.1, 1.0, 5.0, 10.0, and 25.0 µg/mL, and GO-NP-Pt at mixed concentrations of GO 5.0: NP-Pt 0.1 µg/mL, GO 10.0: NP-Pt 1.0 µg/mL, GO 25.0: NP-Pt 5.0 µg/mL, GO 50.0: NP-Pt 10.0 µg/mL, and GO 100.0: NP-Pt 25.0 µg/mL were introduced to the cells for the following 24 h. Next, the BrdU labeling reagent was added and incubated with cells for 24 h. Subsequently, the culture media was removed, the cells were fixed, and the DNA was denatured in one step by adding FixDenat. In the following step, the cells were incubated with anti-BrdU- Fab fragments conjugated with peroxidase (POD) antibodies for 90 min at room temperature. After the removal of the antibody conjugate, the cells were washed, and the substrate solution was added. The reaction product was quantified by measuring absorbance using a scanning multi-well spectrophotometer (Infinite M200, Tecan, Durham, NC, USA) at 370 nm with a reference wavelength of 492 nm and finally expressed as the difference between BrdU-positive and -negative samples, expressed as optical density (OD). All measurements were performed in triplicate.

### 2.4. Cell Viability Assay

Cell viability was evaluated using a 2,3-bis-(2-methoxy-4-nitro-5-sulfophenyl)-2H-tetrazolium-5-carboxyanilide salt (XXT)-based cell viability assay kit (Life Technologies, Taastrup, Denmark). Cells MCF-7, LNCaP, Hela B, HepG2, SW480, HT29, HCT116, and Colo205 were incubated in 96-well plates (5 × 10^4^ cells per well) with hydrocolloids of nanoparticles of GO, NP-Pt, and GO-NP-Pt at the same concentrations as for the proliferation assessment. In the subsequent step, the XTT solution was added to each well and incubated for an additional 3 h at 37 °C. The optical density (OD) of each well was recorded at 450 nm in a scanning multi-well spectrophotometer (Infinite M200, Tecan, Durham, NC, USA). Cell viability was expressed as a percentage (ODtest—ODblank)/(ODcontrol—ODblank), where “ODtest” is the optical density of cells exposed to NP-Pt, GO, and GO-NP-Pt, “ODcontrol” is the optical density of the control sample, and “ODblank” is the optical density of wells without cancer cells.

### 2.5. Cell Morphology

Based on obtained results of the viability and proliferation status of MCF-7, LNCaP, Hela B, HepG2, SW480, HT29, HCT116, and Colo205 cells for cell morphology investigation, a selection was made, and MCF-7, Colo205, and HepG2 were chosen as the most interesting results. Cells from the selected cells line were placed on 6-well plates (1 × 10^5^ cells per well) and treated with NP-Pt (25.0 µg/mL), GO (100.0 µg/mL), and GO-NP-Pt (GO_100_:Pt_25_ µg/mL). After 24 h of incubation with nanoparticles, the cell morphology was recorded under an optical microscope (DM750; Leica Microsystems GmbH, Wetzlar, Germany), using the software package LAS EZ version 2.0. All measurements were performed in triplicate.

The scanning electron microscopy (SEM) analysis of MCF-7, Colo205, and HepG2 cancer cells, treated with (100 µg/mL), NP-Pt (25 µg/mL), and GO-NP-Pt (GO_100_:Pt_25_ µg/mL), was performed by means of a FEI QUANTA 200 electron microscope (Hillsboro, OR, USA). The cell samples were rinsed in phosphate buffered saline (PBS) (0.1 M, pH 7.2; P4417, Sigma), fixed in 2.5% glutaraldehyde (G5882, Sigma) for 1 h, washed twice in 0.1 M PBS (0 01 M, pH 7.2; P4417, Sigma), and placed on aluminum SEM stubs. The SEM stubs were kept in a moist atmosphere for 1 h, washed in PBS (0.1 M, pH 7.2; P4417, Sigma), post-fixed in 1% osmium tetroxide (75632, Sigma) for 1 h, rinsed in distilled water, and dehydrated in graded ethanol. After critical-point drying with liquid CO_2_ in a vacuum apparatus (Polaron CPD 7501, Quorum Technologies, Newhaven, East Sussex, UK) and coating with gold-palladium (JEE-4C, JEOL Ltd., Tokyo, Japan), the samples were inspected by SEM at 1 keV (FEI QUANTA 200).

### 2.6. Apoptosis Assay

Apoptosis was evaluated using the Alexa Fluor^®^ 488 Annexin V/Dead Cell Apoptosis Kit with Alexa Fluor 488 Annexin V and propidium iodide (PI) for flow cytometry (Life Technologies, Carlsbad, CA, USA). The MCF-7, Colo205, and HepG2 cells (1 × 10^5^ cells per well) were incubated for 24 h. Subsequently, the medium was removed, and GO (100 µg/mL), NP-Pt (25 µg/mL), and GO-NP-Pt (GO_100_:Pt_25_ µg/mL) in the culture medium were added to the cells and incubated for an additional 24 h. The positive control was prepared as described in Jaworski et al. [26] After this, the MCF-7, Colo 205, and HepG2 cells were harvested, washed in cold PBS, transferred to tubes, and stained according to the Annexin V/PI staining protocol (Life Technologies). Cancer cells were analyzed by flow cytometry (FACSCalibur, Becton Dickinson, Franklin Lakes, NJ, USA), measuring the fluorescence emission at 530 nm and 575 nm (or equivalent), using excitation at 488 nm. Positive cells were identified on the basis of the fluorescence intensity of Annexin V-Alexa Fluor 488 (early stage of apoptosis) or PI (end stage of apoptosis and necrosis). Data were analyzed using the Cell Quest Pro software ver. 5.1 (Becton Dickinson), and the regions were set on the basis of positive and negative control samples. 

### 2.7. Gene Expression

#### 2.7.1. Isolation of Total RNA

For the isolation of total RNA, MCF-7, Colo205, and HepG2 cells (1 × 10^5^ cells per well) were incubated for 24 h. Subsequently, the medium was removed, and GO (100 µg/mL), NP-Pt (25 µg/mL), and GO-NP-Pt (GO_100_:Pt_25_ µg/mL) in the culture medium were added to the cells and incubated for an additional 24 h. Total RNA was isolated using a PureLink^®^ RNA Mini Kit (Ambion™ Life Technologies, Foster City, CA, USA). The resulting cell pellet was resuspended in lysis buffer containing 1% 2-mercaptoethanol, and subsequently, the frozen metal balls were added to the probe and homogenized in a TissueLyser ball mill (Qiagen, Germantown, MD, USA) for 5 min at 50 Hz. The homogenate was centrifuged at 12,000× g. The supernatant, containing total RNA, was transferred into a new tube, and one volume 70% ethanol was added into each volume of cell homogenate, following the manufacturer’s instructions. Total RNA was eluted in a volume of 50 µL RNase-free water and stored at −80 °C. The isolated RNA was measured using a NanoDrop 2000 spectrophotometer (Thermo Scientific, Wilmington, DE, USA). The cDNA was synthesized with a cDNA High Capacity Reverse Transcription Kit (AppliedBiosystems, Foster City, CA, USA) to reverse-transcript the mRNA to cDNA, using 2200 ng per reaction. The obtained cDNA was measured using a NanoDrop 2000 spectrophotometer and stored for further analysis at −20 °C. 

#### 2.7.2. Real-Time PCR 

The ∆∆Ct method was used to determine the expression of mRNA, using real-time PCR:

∆∆CT = ∆CT test sample − ∆CT calibrator sample

The reaction was carried out using 48-well plates and the Luminaris Color HiGreen reagents qPCR Master Mix (Thermo Fisher Scientific, Waltham, MA, USA); 100 ng of cDNA were used for each reaction. The following genes were examined: *caspase-3* and *proliferating cell nuclear antigen* (*PCNA)*. The primers used for this procedure are presented in Table 1. Glyceraldehyde-3-phosphate dehydrogenase (GPDH) was used as the reference house-keeping gene. The reaction conditions were set as specified by the manufacturer, and each sample was analyzed in duplicate. The procedure was conducted using a StepOnePlus™ Real-Time PCR System. 

### 2.8. Statistical Analysis

Data were analyzed using two-way analysis of variance Graph Pad Prism B ver. 8 (GraphPad Software, San Diego, CA, USA). Differences between groups were tested using Tukey’s multiple range tests. All mean values are presented with standard deviations.

## 3. Results

### 3.1. Characterization of Nanocolloids

#### 3.1.1. Scanning Transmission Electron Microscopy

**Figure 1:**
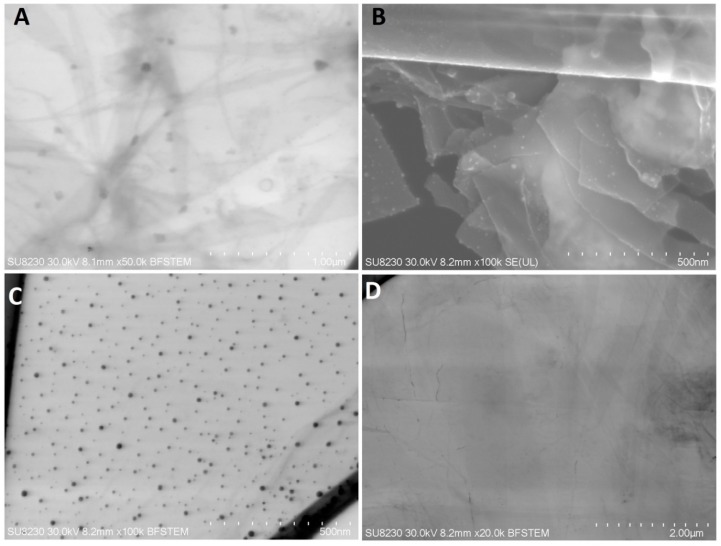
Scanning transmission electron microscopy images of (**A**,**B**) nanocomplexes of graphene oxide and platinum nanoparticles, (**C**) platinum nanoparticles, and (**D**) graphene oxide. Scale bars: 500 nm–2 µm. Abbreviations: GO-NP-Pt—nanocomplexes of graphene oxide and platinum nanoparticles, NP-Pt—platinum nanoparticles, GO—graphene oxide.

The NP-Pt had a regular round shape, with a particle diameter from 2 to 19 nm (Figure 1C). Agglomeration of NP-Pt colloids was not detected. The GO platelets were observed as a single or few layers, with an irregular shape with jagged edges (Figure 1D). The GO-NP-Pt complexes were observed as a few layers of GO platelets covered irregularly with round Pt nanoparticles (Figure 1A,B).

#### 3.1.2. Fourier Transform Infrared (FTIR) Spectroscopy

**Figure 2:**
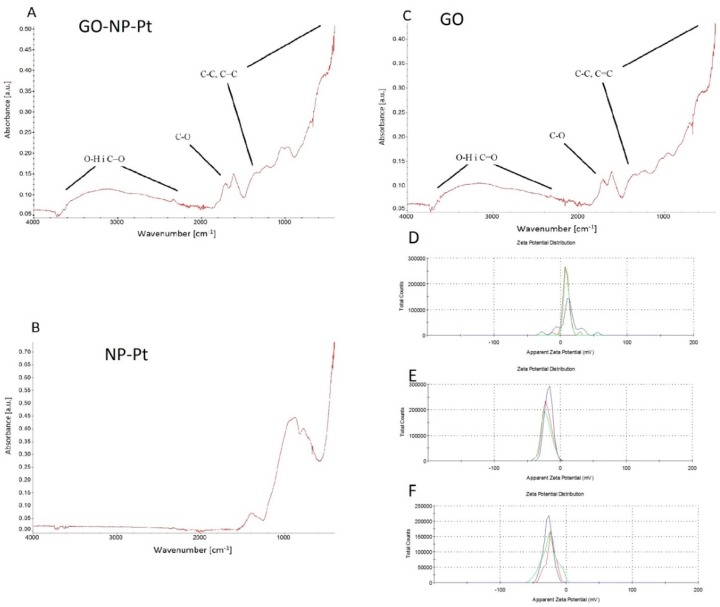
FTIR spectrum and ζ-potential of nanocomplexes of (**A**,**D**) graphene oxide and platinum nanoparticles, (**B**,**E**) platinum nanoparticles, and (**C**,**F**) graphene oxide. Abbreviations: GO-NP-Pt—nanocomplexes of graphene oxide and platinum nanoparticles, NP-Pt—platinum nanoparticles, GO—graphene oxide.

The FTIR spectrum of GO-NP-Pt and GO showed signals at wavenumbers of 1700 cm^−1^, 1650 cm^−1^, and 1200 cm^−1^, which are associated with C=O, C=C, and C–O bonds of GO, respectively. The high signal starting from around 1000 cm^−1^ corresponds to C–C and C–H bonds. Finally, the broad signal around 3500 cm^−1^ is a result of the O–H bonds of hydroxyl groups in GO (Figure 2A–C).

#### 3.1.3. ζ-Potential Measurements

The ζ-potential for GO-NP-Pt was −23.7 mV and it was the most stable colloid of nanoparticles (Figure 2D). The ζ-potential was highest for Pt-NP (−19.0 mV) (Figure 2E) and lowest for GO (−42.0 mV (45%), −21.6 (28.8%) (Figure 2F).

### 3.2. Proliferation Assay by BrdU Incorporation

**Figure 3:**
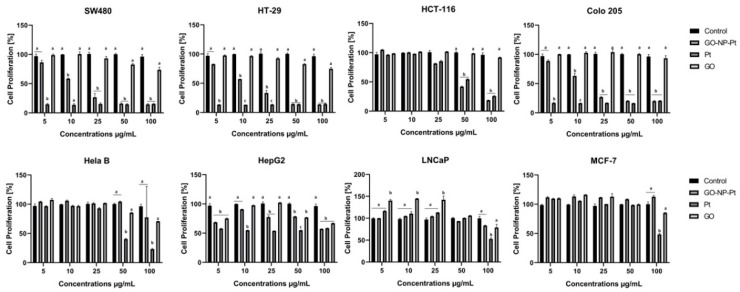
Effects of nanocomplexes of graphene oxide and platinum nanoparticles, platinum nanoparticles, and graphene oxide. on cell proliferation. Notes: Different lowercase letters (a and b) within columns indicate significant differences between the concentrations (*p* < 0.05). Abbreviations: GO-NP-Pt—nanocomplexes of graphene oxide and platinum nanoparticles, NP-Pt—platinum nanoparticles, GO—graphene oxide.

The synthetic analogue of thymidine, called bromodeoxyuridine (BrdU), is implemented into the DNA helix during DNA synthesis. The decreased level of BrdU was equal to the decreased cell proliferation. The most sensitive to antiproliferative activity of nanoparticles were colorectal cancer cells from the cell lines SW480, Colo 205, and HTC116. The highest inhibition of proliferation was observed in Colo 205 cells. The decreased levels of proliferation in SW480, Colo205, HTC116, and HT29 after treatment with nanocomplexes of GO-NP-Pt were similar to the results after NP-Pt treatment (Figure 3).

The opposite results were obtained with the cell lines liver cancer HepG2, human breast cancer MCF-7, adenocarcinoma LNCaP, and human cervical Hela B cell lines. The most resistant to GO-NP-Pt proliferation inhibition were MCF-7 and Hela B cell lines. In HepG2 and LNCaP cell lines, the proliferation regression was similar, but HepG2 liver cancer cells were more sensitive (Figure 3).

### 3.3. Cell Viability Assay

**Figure 4:**
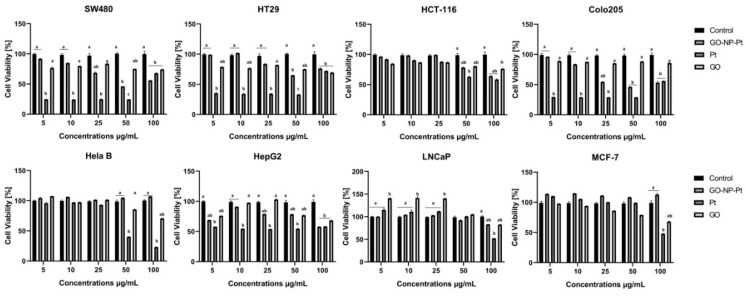
Effects nanocomplexes of graphene oxide and platinum nanoparticles, platinum nanoparticles, and graphene oxide on cell viability. Notes: Different lowercase letters (a and b) within columns indicate significant differences between the concentrations (*p* < 0.05). Abbreviations: GO-NP-Pt—nanocomplexes of graphene oxide and platinum nanoparticles, NP-Pt—platinum nanoparticles, GO—graphene oxide.

The XTT cell viability assay is based on the ability of reducing the tetrazolium salt XTT into orange formazan by metabolically active (live) cells. The results showed that the highest reduction of viability after GO-NP-Pt treatment was observed in the cell lines Colo205 and HepG2 (Figure 4). The GO treatment at concentrations between 5–100 µg/mL had a minor impact on cell viability. The NP-Pt treatment at the highest tested concentration was the most toxic treatment for all types of investigated cancer cells (Figure 4). The HepG2 cell line showed sensitivities at the viability assessment for GO, NP-Pt, and GO-NP-Pt treatments similar to those in the proliferation activity investigation. In both investigations, HepG2 cells showed a 50% reduction of cancer cell viability and proliferation at the highest concentration of GO-NP-Pt (GO_100_:Pt_25_ µg/mL).

### 3.4. Cell Morphology

Based on proliferation activity and viability, the cell lines Colo205, HepG2, and MCF-7 were selected for cell morphology investigations.

**Figure 5:**
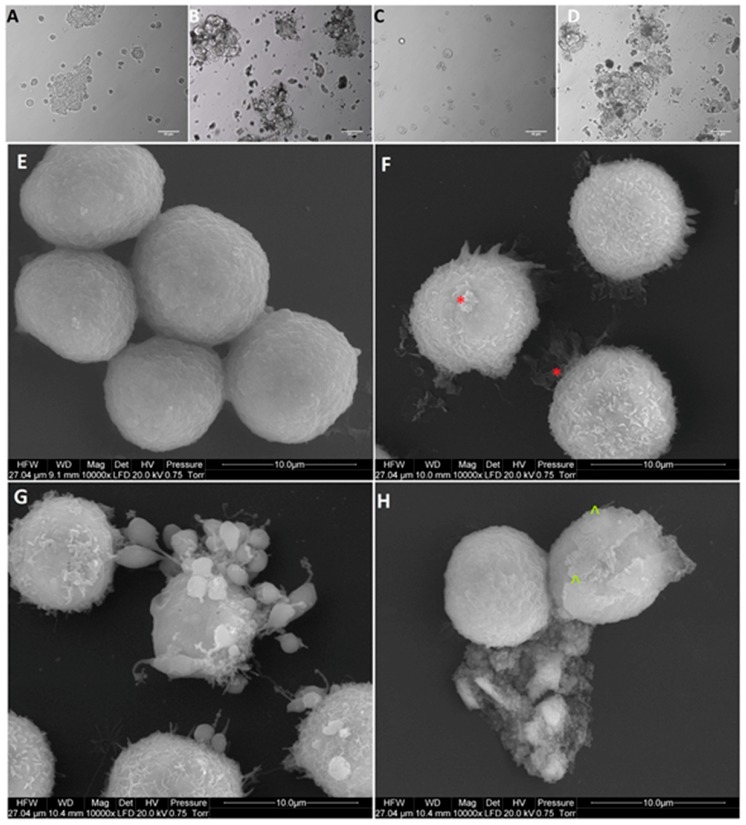
Morphology of Colo205 colorectal cancer cells. (**A**,**E**) untreated cells (control group), (**B**,**F**) cells treated with nanocomplexes of graphene oxide with platinum nanoparticles (GO-NP-Pt), (**C**,**G**) cells treated with platinum nanoparticles (NP-Pt) t, (**D**,**H**) cells treated with graphene oxide (GO). Red *—point on GO-NP-Pt at cell membrane. Green **^**—point on GO at cell membrane. (**A**–**D**) Light optical microscopy. Scale bars: 50 µm. (**E**–**H**) Scanning electron microscopy. Note: Scale bars: 10 µm. Abbreviations: GO-NP-Pt—nanocomplexes of graphene oxide and platinum nanoparticles, NP-Pt—platinum nanoparticles, GO—graphene oxide.

**Figure 6:**
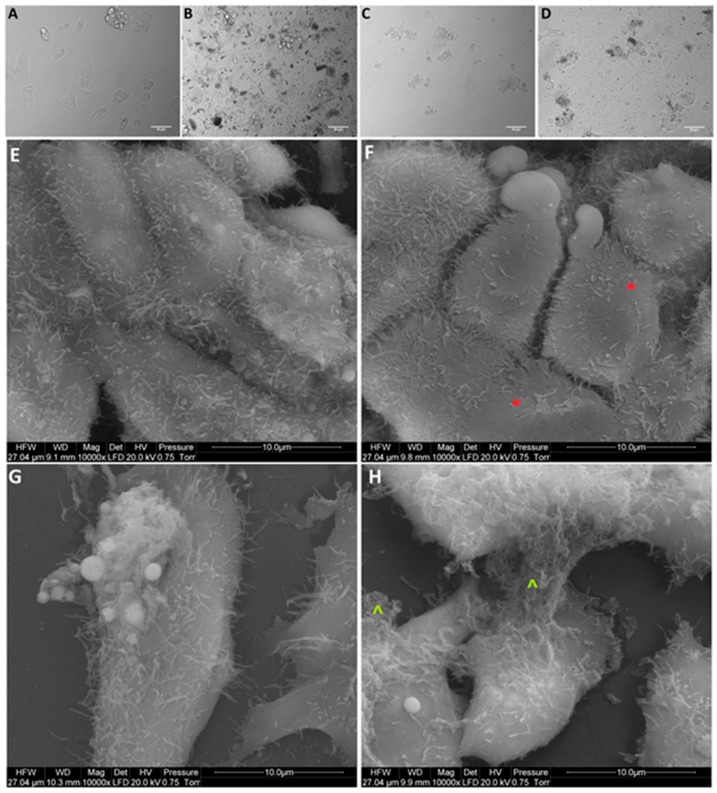
Morphology of HepG2 liver cancer cells. (**A**,**E**) untreated cells (control group), (**B**,**F**) cells treated with nanocomplexes of graphene oxide and platinum (GO-NP-Pt), (**C**,**G**) cells treated with platinum nanoparticles (NP-Pt), (**D**,**H**) cells treated with graphene oxide (GO). Red *—point on GO-NP-Pt at cell membrane. Green **^**—point on GO at cell membrane. (**A–D**) Light optical microscopy. (**E–H**) Scanning electron microscopy. Notes: A–D scale bars 50 µm, E–H scale bars 10 µm. Abbreviations: GO-NP-Pt—nanocomplexes of graphene oxide and platinum nanoparticles, NP-Pt—platinum nanoparticles, GO—graphene oxide.

**Figure 7:**
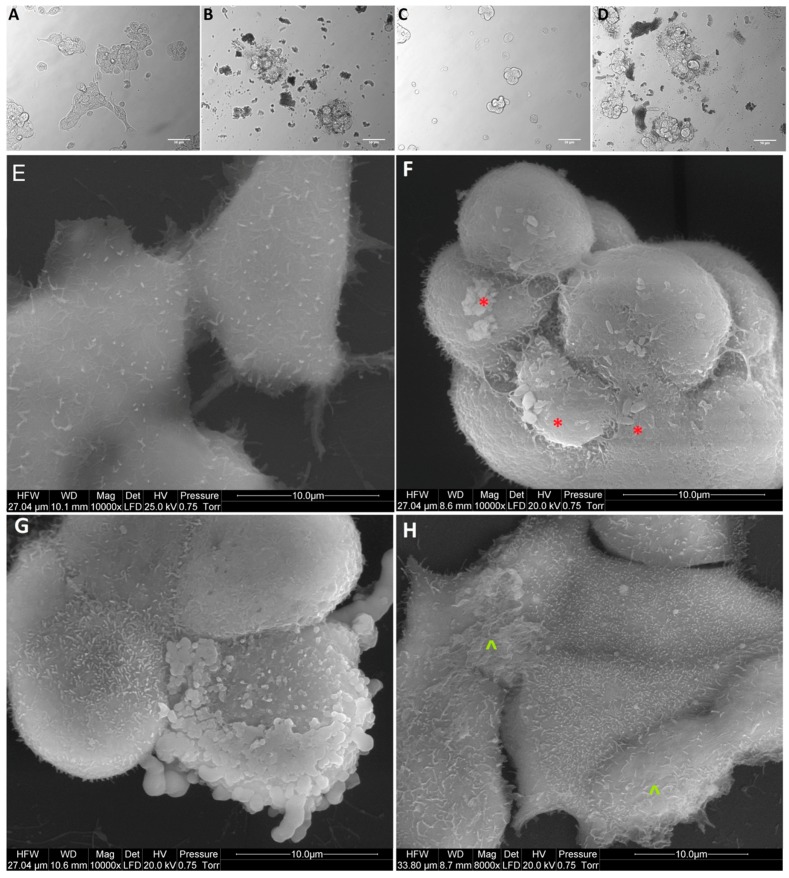
Morphology of MCF-7 breast cancer cells. (**A**,**E**) untreated cells (control group), (**B**,**F**) cells treated with nanocomplexes of graphene oxide with platinum nanoparticles (GO-NP-Pt), (**C**,**G**) cells treated with platinum nanoparticles (NP-Pt), (**D**,**H**) cells treated with graphene oxide (GO). Red *—point on GO-NP-Pt at cell membrane. Green **^**—point on GO at cell membrane. (**A–D**) Light optical microscopy. (**E–H**) Scanning electron microscopy. Notes: A–D scale bars 50 µm, E–H scale bars 10 µm. Abbreviations: GO-NP-Pt—nanocomplexes of graphene oxide and platinum nanoparticles, NP-Pt—platinum nanoparticles, GO—graphene oxide.

After treatment with GO, NP-Pt, and GO-NP-Pt, the selected cells showed reduced cell density and deformation of cell membranes compared to the non-treated control group (Figure 5, Figure 6 and Figure 7). The GO-NP-Pt (Figure 5B,E; Figure 6B,E; Figure 7B,E) were attached to the cell body and had a high affinity to the cell membranes. The NP-Pt caused major deformation of the cell structure, including cell membranes, and reduced the length of cell protrusions (Figure 5C,F; Figure 6C,F; Figure 7C,F). Scanning electron microscope (SEM) images (Figure 5, Figure 6 and Figure 7) showed that GO platelets had a high affinity to the cell membrane and caused minor deformation of the membrane (Figure 5D,G; Figure 6D,G; Figure 7D,G) compared to the cells from the control group (Figure 5A,D; Figure 6A,D; Figure 7A,D).

### 3.5. Apoptosis Assay

**Figure 8:**
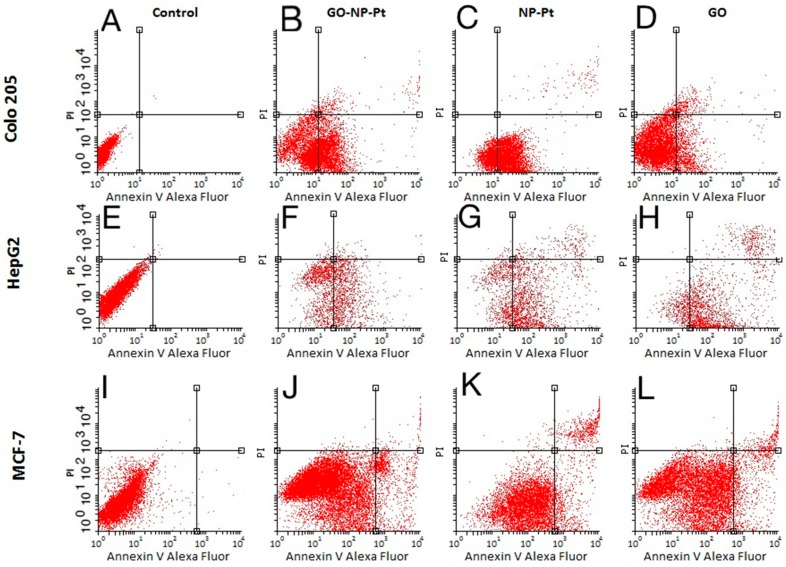
Apoptosis assay. Flow cytometry Annexin V-Alexa Fluor^®^ 488/propidium iodide (PI) assay. Scatter diagrams of Colo205 (**A**–**D**), HepG2 (**E**–**H**), and MCF-7 (**I**–**L**) cells exposed to nanocomplexes of graphene oxide with platinum nanoparticles (**B**,**F**,**J**), platinum nanoparticles (**C**,**G**,**K**), and graphene oxide (**D**,**H**,**L**). Control group—non treated cells (**A**,**E**,**I**). Abbreviations: GO-NP-Pt—nanocomplexes of graphene oxide and platinum nanoparticles, NP-Pt—platinum nanoparticles, GO—graphene oxide.

The apoptosis assay based on annexin V/PI is an effective method for detecting the type of cell death via detection of phosphatidylserine by annexin V on cell membranes. The level of apoptosis- positive cells at control non treated group showed insignificant level of apoptosis and necrosis (Figure 8A,E,I). The level of apoptosis-positive cells was the highest after treatment with GO-NP-Pt nanocomplexes in the MCF-7 cell line (Figure 8J). The GO platelets significantly increased apoptotic cell death in HepG2 and MCF-7 cells (Figure 8H,L). The Colo 205 cells were most resistant for activation of apoptosis under GO treatment (Figure 8D). The degree of necrosis was highest in MCF-7 cells after GO treatment (Figure 8L), while the number of necrotic cells after NP-Pt treatment was significantly lower than in the control group in all investigated cell lines (Figure 8C,G,K).

### 3.6. Gene Expression

The level of mRNA expression of *caspase-3* increased after GO-NP-Pt treatment of Colo205 cells (Table 2). The difference was significant compared to the control group and GO and NP-Pt treatments. The GO-NP-Pt treatment of HepG2 and MCF-7 caused insignificant decreases in *caspase-3* expression compared to the control group. The cell proliferation status was verified by the evaluation of mRNA expression of *PCNA*, which showed a tendency towards decreasing after all treatments compared to the control group. The cell lines Colo205 and MCF-7 were most sensitive for GO-NP-Pt treatment, where the mRNA expression of PCNA was significantly decreased. The mRNA expression of PCNA after GO-NP-Pt treatment of HepG2 cells decreased, although the differences between the GO-NP-Pt-treated group and the non-treated control group were insignificant. The Colo205 cancer cells showed the most significant decrease of *PCNA* expression after NP-Pt treatment compared to the non-treated cells (control group).

## 4. Discussion

Nanotechnology provides advanced techniques for the detection of cancer cells, the delivery of anticancer drugs, or the activation of programmed cell death by nanoparticles. Nanoparticles have unique physiochemical properties such as nanometric size, large surface-to-mass ratio, and high reactivity with biological structures. The NP-Pt have been considered as an alternative to bulk Pt anticancer agents, showing cytotoxic activity against different types of cancer cells [16,17,18,19,20,21,26]. The main mechanism of NP-Pt action is related to the high catalytic activity of Pt and their ability to interact with cell components, including genetic material [17,19,20,21,27]. The NP-Pt induce programmed cell death by apoptosis after direct interaction with cancer cell DNA helix [16,18]. However, the possibility of aggregation of NP-Pt and uncontrolled biodistribution are the major drawback of its usage in anticancer therapy.

In the present work, the synthesized complex of GO and NP-Pt was composed of GO multilayers decorated with well-dispersed NP-Pt on the GO surface. The surface of graphene oxide and its functionalization are significant for its physicochemical features and thereby important in the anticancer assessment of the GO-NP-Pt complex. The present results may only be representative for the used complex. The complex of GO-NP-Pt was created by self-organization of GO and Pt, induced by sonication. The FTIR analysis of GO-NP-Pt did not show any changes in the chemical bonding on the surface of GO platelets coated with NP-Pt compared to the raw GO platelets, indicating that the type of the connection between GO platelets and NP-Pt is based on non-chemical bonding such as van der Waals forces. Wen et al. [28] also reported that silver nanoparticles were able to impinge the cytoskeletal structures, not by chemical reactions, but by van der Waals forces. Our findings are consistent with the previously published data where negatively charged nanoparticles had stronger harmful effects on cells than cationic particles [29]. The obtained **ζ**-potential measurements of GO-NP-Pt showed that the noncomplex had a higher anionic charge than NP-Pt, which probably decreased its cytotoxic effect against cancer cells compared to NP-Pt treatment. The internalization of GO-NP-Pt is related to the membrane tension of cancer cells and the size of delivered nanoparticles [30]. The size of investigated complex of GO-NP-Pt was close to the size of GO platelets. Regarding to previous published data by Linares et al. [31], the main mechanism of cellular GO-NP-Pt internalization was the endocytosis, but to clarify this mechanism the future follow-up research is needed. These results are probably related to GO-NP-Pt electrical charge disruptions compared to NP-Pt, affecting the cell response to nanoparticles. The charge, size, shape, and the chemical composition have an impact on the cytotoxicity of nanoparticles [32]. In previous studies, NP-Pt had a size-dependent toxicity to cancer and normal cells as well as tissues [15,33]. 

The goal of GO functionalization by NP-Pt was to enhance the anticancer activity of GO and NP-Pt. Zhang et al. [24] also obtained NP-Pt-loaded GO nanostructures by placing well-dispersed NP-Pt onto folic acid-modified GO platelets, but the chemical reactions of this synthesis were more complicated and involved activity of poly (allylamine hydrochloride). The catalytic activity of NP-Pt could be limited by the addition of chemical or biological stabilizers to prevent NP-Pt agglomeration. However, the usage of GO as an anchoring platform helps to prevent agglomeration without losing its anticancer properties. The present study showed that the density of Pt coating was irregular, but NP-Pt located on the specific parts of GO platelets, especially on the edges as well as on the wrinkles of graphene oxide. 

In the present study, we observed decreased proliferation and viability in the majority of the inspected cancer cell lines. The results of the viability assessment of GO-NP-Pt, NP-Pt, and GO showed that the best anticancer efficiency was observed after GO-NP-Pt at the concentration of GO_100_:Pt_25_ µg/mL against HepG2 and MCF-7 cells. The proliferation status of SW480, HT29, and Colo 205 cancer cell lines was significantly decreased after GO-NP-Pt treatment with the highest concentration (GO_100_:Pt_25_ µg/mL). However, the MFC-7 cell line was the most resistant one to GO-NP-Pt at all concentrations. The results, also showed that cytotoxicity of NP-Pt located on GO platelets, decreased compared to the bare NP-Pt. The synthesis of nanocomplexes of GO-NP-Pt was beneficial for limitation of uncontrolled released of NP-Pt in a biological system and helps to decrease the possible side effects of NP-Pt. Similar results were obtained by Zhang et al. [24], where MCF-7 cells showed strong GO-NP-Pt resistance. In these studies, the authors also pointed out that GO acted bidirectionally as a stabilizer and reducing agent to obtain a high quality of well-dispersed NP-Pt on the GO surface. Moreover, GO platelets are biocompatible materials, with minor effects on cell structure and metabolism [34], but still have an extremely high capacity for cell membrane attachment [35]. The HepG2 cell line showed the insignificant changes in mRNA expression of *PCNA.* The results indicated that the characteristic type of cell grow of HepG2 cell line, had an impact on a possible attachment of GO-NP-Pt to the cell membrane. The lower GO-NP-Pt attachment to the cell membrane reduced cytotoxic effect of GO-NP-Pt nanocomplex and had no impact on the mRNA expression of *PCNA*. Furthermore, at the gene expression profile of HepG2 cell, the mRNA level of MRP2—multidrug resistance protein 2 is overexpressed and can lead to developing chemoresistance [36] for anticancer activity of Pt atoms. The present results in terms of cell morphology showed that GO platelets had an extremely high affinity to cell membranes in all experimental groups, with a minor degree of deformation. On the other hand, NP-Pt treatment caused major deformations of Colo 205, HepG2, and MCF-7 cell structure with loss of plasma membrane integrity. Nanocomplexes of GO-NP-Pt at the highest concentration of GO_100_:Pt_25_ µg/mL showed a high affinity to cell membranes and also caused cell body deformation. Moreover, the high affinity of GO-NP-Pt to the cell membranes was characteristic for all types of inspected cell lines, which indicates that nanoparticles can inhibit cellular functions of any type of cancer cell lines. The main mechanism of interaction between GO-NP-Pt and the cell surface was probably based on interactions between nanoparticles and structural proteins through electrostatic forces. Our results demonstrate that the level of mRNA expression of *caspase-3* increased significantly after GO-NP-Pt treatment only in the Colo 205 cell line compared to the non-treated cells (control group). A previous study [37] showed that GO combined with metal nanoparticles caused up-regulation of the mRNA levels of *caspase-3*, *caspase-9*, and *Bax* genes and activated programmed cell death. Analysis of mRNA expression of *PCNA* showed that GO-NP-Pt caused a significant reduction of the *PCNA* gene expression level of the cell lines Colo 205 and MCF-7. 

There are three main cancer treatment strategies: surgery, chemotherapy, and radiation. The major drawbacks of all these treatments are the lack of selectivity between cancer and non-cancer cells and/or incomplete eradication of cancer cells. Nanoparticles of GO and NP-Pt are of interest for the development of cancer treatments because of their unique physiochemical and biological properties [8,9,10,16,17,18,19,20,21]. However, the accumulation of NP-Pt into cells and body tissue after in-vivo administration is also one of the drawbacks for future clinical applications. The results of biodistribution and toxicity evaluation showed that NP-Pt were accumulated in the liver and spleen, but small amounts of Pt were also detected in other tissues of mice 24 h after administration. However, after prolonged administration, NP-Pt were mainly retained in the liver and spleen, and only negligible amounts were found in the other tissues [23]. Similar results were obtained after GO in-vivo administration by intravenous injection, where GO platelets were also accumulated in the liver [34]. The results of biodistribution evaluation of GO also showed that GO platelets had a non-systemic toxicity, but a tendency to accumulate and aggregate in the vicinity of the injection sites [38]. GO is a biocompatible material with surface that is easy to functionalize with nanoparticles or bioactive molecules [13,14,15,35]. The goal of using the drug deliver carriers is the effectiveness, selectivity and safety of drug administration. GO platelets are biocompatible, which means that GO is able to perform an appropriate host response without undesirable local or systemic effects. The biocompatibility of GO increases the local ability of NP-Pt to perform their anticancer activity. Moreover, GO has an ability to cross the cell membrane structure and delivered GO platelets into cancer cells, partly without involving transporting proteins [8]. These features can limit the development of multiple drug resistance in cancer cells. The accumulation at injection sites may be useful for effective direct intratumor injection of GO functionalized with NP-Pt and to increase local anticancer activity of Pt. 

## 5. Conclusions

The Pt release from GO-NP-Pt complexes restricted the NP-Pt activity to the local structure. The GO platelets were the efficient delivery platform for NP-Pt and presented a high affinity to Colo 205, HepG2, and MCF-7 cancer cell membranes, confirming the benefits of using GO platelets as an anchoring platform for NP-Pt. The application of GO-NP-Pt complexes decreased cell viability and proliferation and activated apoptosis in the Colo205 cancer cell line. The proliferation status was decreased after GO-NP-Pt treatment in HepG2 and MCF-7 cell lines, but in HepG2 and MCF-7 cells, programmed cell death was not activated at the gene expression level. These results indicate that the nanocomplex of GO-NP-Pt may be useful in cancer therapy, but the interaction between target cancer cell lines and GO-NP-Pt and potential side effects must be elucidated by in-vivo research. 

## Figures and Tables

**Table 1:** Primer sequences for the investigated genes.

Target Gene	Forward Primer	Reverse Primer
*Caspase-3*	CAAACTTTTTCAGAGGGGATCG	GCATACTGTTTCAGCATGGCAC
*PCNA*	AGGCACTCAAGGACCTCATCA	GAGTCCATGCTCTGCAGGTTT
*GPDH*	ACATCCCCTCACCAAT AACAAC	TAGCCAAATCATACTGCTCGTC

Abbreviations: *PCNA*—proliferating cell nuclear antigen; *GPDH*—glyceraldehyde-3-phosphate dehydrogenase.

**Table 2:** *Caspase-3* and *PCNA* expression profile in Colo205, HepG2, and MCF-7 cancer cell lines after GO-NP-Pt, NP-Pt, and GO treatments.

Level of mRNA Expression		Groups				ANOVA *p*-Value	SE-Pooled
*Gene*	Cell Line	Control	GO-NP-Pt	NP-Pt	GO		
*Caspase-3*	Colo205	0.738 ^a^	1.222 ^c^	0.555 ^b^	0.756 ^a^	0.0036	0.1316
	HepG2	0.742 ^a^	0.727 ^a^	0.379 ^b^	0.321 ^b^	0.0122	0.1567
	MCF-7	0.832	0.692	0.897	1.067	0.0396	0.1874
*PCNA*	Colo205	1.001 ^a^	0.774 ^b^	0.579 ^c^	0.832 ^a^	0.0021	0.0689
	HepG2	1.253	0.991	0.780	0.886	0.5606	0.3351
	MCF-7	1.016 ^a^	0.588 ^b^	0.837 ^a^	0.994 ^a^	0.0491	0.1378

Abbreviations: *PCNA*—proliferating cell nuclear antigen. Notes: ^a,b,c^ Values within rows with different superscripts are significantly different at *p* < 0.05. Values were normalized to the housekeeping gene *GPDH.* Abbreviations: GO-NP-Pt—complexes of graphene oxide and platinum nanoparticles, NP-Pt—platinum nanoparticles, GO—graphene oxide; ANOVA—analysis of variance, SE—standard error.
